# Comparative influence of genetics, ontogeny and the environment on elemental fingerprints in the shell of *Perna canaliculus*

**DOI:** 10.1038/s41598-019-44947-0

**Published:** 2019-06-12

**Authors:** Craig R. Norrie, Brendon J. Dunphy, Norman L. C. Ragg, Carolyn J. Lundquist

**Affiliations:** 1 Institute of Marine Science, University of Auckland, Auckland, New Zealand; 2 School of Biological Sciences, University of Auckland, Auckland, New Zealand; 3 Cawthron Institute, Nelson, New Zealand; 4 National Institute of Water and Atmospheric Research (NIWA), Hamilton, New Zealand

**Keywords:** Ecology, Zoology, Ecology

## Abstract

The trace elemental composition of biogenic calcium carbonate (CaCO_3_) structures is thought to reflect environmental conditions at their time of formation. As CaCO_3_ structures such as shell are deposited incrementally, sequential analysis of these structures allows reconstructions of animal movements. However, variation driven by genetics or ontogeny may interact with the environment to influence CaCO_3_ composition. This study examined how genetics, ontogeny, and the environment influence shell composition of the bivalve *Perna canaliculus*. We cultured genetically distinct families at two sites *in situ* and in the laboratory. Analyses were performed on shell formed immediately prior to harvest on all animals as well as on shell formed early in life only on animals grown in the laboratory. Discriminant analysis using 8 elements (Co, Ti, Li, Sr, Mn, Ba, Mg, Pb, Ci, Ni) classified 80% of individuals grown *in situ* to their family and 92% to growth site. Generalised linear models showed genetics influenced all elements, and ontogeny affected seven of eight elements. This demonstrates that although genetics and ontogeny influence shell composition, environmental factors dominate. The location at which shell material formed can be identified if environmental differences exist. Where no environmental differences exist, genetically isolated populations can still be identified.

## Introduction

The trace elemental composition of biogenic calcium carbonate (CaCO_3_) structures such as fish otoliths, bivalve shells, and gastropod statoliths has been the focus of a number of studies over recent decades^1–4^. The composition of these structures has been shown to reflect a number of environmental parameters. Analysis of the trace elemental composition of carbonate structures formed early in an individual’s life can therefore be used to infer the water mass in which they formed and provide information on natal locations and population connectivity^5–7^. This technique of reconstructing animal movements from calcium carbonate structures is known as trace elemental fingerprinting^8,9^.

Implicit in the use of the trace elemental fingerprinting is the assumption that these calcified structures reflect the properties of the water mass in which they were formed. Thus, there is a significant body of literature investigating the relationship between environmental parameters and the trace elemental composition of biogenic CaCO_3_. In bivalves the trace elemental composition of shell material has been shown to reflect temperature, salinity, pH, and diet^6,7,10–12^. The relationships between physical variables and the trace elemental composition of CaCO_3_ structures, however, is not always consistent. For example, positive relationships, negative relationships, and no relationships at all have been found between temperature and trace element:calcium (*TE*:Ca) ratios in in bivalve shells^13–15^. This suggests that other factors intrinsic to individuals influence the trace elemental composition of CaCO_3_ structures.

Although the influence of genetic variation on the incorporation of trace elements into bivalve shell has not been previously investigated, laboratory studies have shown that the trace elemental fingerprint in fish otoliths is a result of interactions between the environment and the genetic origin of individuals^16–18^. Understanding whether genetic variation can affect the elemental composition of bivalve shell is essential before inferences on the conditions under which they formed can be made and ecologically relevant information obtained. If the incorporation of trace elements to shell material is dominated by genetics, the use of trace elemental fingerprinting to reconstruct animal movements may be limited. Similar elemental fingerprints may be misinterpreted as being caused by shell forming at the same location rather than due to genetic similarities. This would be of particular concern within metapopulations where a lack of genetic heterogeneity and diversity results in homogeneous trace elemental fingerprints in individuals from a number of spatially separated sub-populations.

Although laboratory studies provide insight as to how the incorporation of elements into CaCO_3_ structures may vary under tightly controlled conditions, understanding how a broad suite of elements varies under natural conditions is essential for reconstructing animal movements. If both genetic and environmental variation drive the incorporation of elements into shell material, these could enhance differences between populations and improve the utility of this method for determining population connectivity^17^. Regardless of genetic effects if environmental differences between sites exert dominant control over the incorporation of trace elements into shell, the use of trace elemental fingerprinting will remain useful regardless of the genetic relationships between populations.

In addition to biological variation driven by genetic differences, it is also likely that ontogenetic changes in animals will modulate how trace elements are incorporated into biogenic CaCO_3_. In fish otoliths and bivalve shells, age can affect the response to environmental variables^12,16,19^. It is important that any ontogenetic influences are known before inferences on changes in the environment as an animal grows can be determined. If this is the case, it is important that differences in trace elemental fingerprints between locations are greater than any ontogenetic differences to avoid complications in the development of reference fingerprints^20^.

The aim of this study was to determine the influence of the genetic lineage of individuals, the location at which they grew, and ontogeny on the trace elemental composition of the shell of the bivalve *Perna canaliculus* Gmelin 1791 (Greenshell^TM^ mussel). We set out to answer the following three questions: (1) Are environmental differences between sites or genetic differences between families responsible for the trace elemental composition of *P. canaliculus* grown at two separate sites? (2) Does the trace elemental composition of genetically distinct, *P. canaliculus* families vary when grown under identical laboratory conditions? (3) Finally, as an animal grows, what impact does this have on the trace elemental composition of its shell? Using animals from a commercial selective breeding programme, individuals from known genetic lines were studied in both *in situ* and laboratory trials. Variation in the trace elemental composition of shell material was analysed using laser ablation inductively coupled plasma mass spectrometry.

## Results

Ten trace elements (Li, B, Mg, Ti, Mn, Co, Cu, Sr, Ba, Pb) in addition to calcium were consistently detectable in shells of *Perna canaliculus* grown in the field and were therefore considered in further statistical analyses. The most abundant of the elements was strontium followed by magnesium. The least abundant elements were cobalt and lead (Table S4).

### *In situ* trial

#### Quadratic DFA

**Figure 1:**
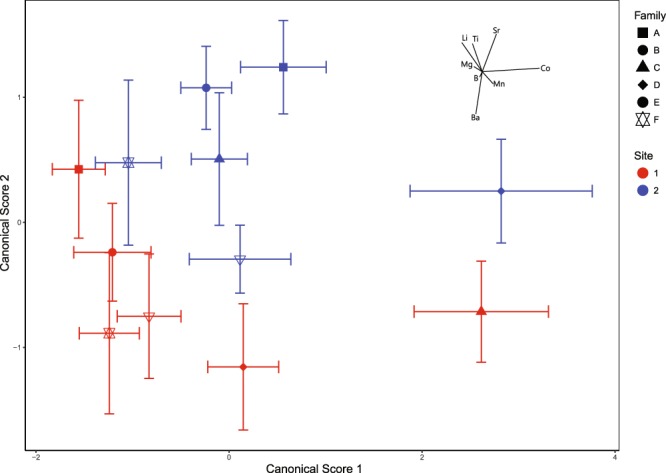
Mean and 95% confidence intervals of canonical scores from the quadratic discriminant function analysis of Co:Ca, Ti:Ca, Li:Ca, Sr:Ca, Mn:Ca, Ba:Ca, Mg:Ca, and B:Ca ratios in the shell of six families of adult *P. canaliculus* cultured at two different sites in the Marlborough Sounds, New Zealand.

Results of the quadratic discriminant function analysis (QDFA) stepwise variable selection procedure revealed that the inclusion of eight trace element:calcium (*TE*:Ca) ratios into the classification model (Co:Ca, Ti:Ca, Li:Ca, Sr:Ca, Mn:Ca, Ba:Ca, Mg:Ca, B:Ca) resulted in correct classification of individuals to their growth site:family combination with 78% accuracy (Tables 1 and 2). In this QDFA individuals were assigned to their growth site with 92% accuracy and their family with 80% accuracy (Table 1).The inclusion of the next most significant element (Pb) increased site:family classification success by only 0.9% (Table 2); therefore only these eight elements were used in further statistical analyses. Additionally, the inclusion of lead into the QDFA resulted in a drop in classification rates to growth site and only a marginal increase in classification to the correct family (Table 2). Plots of means and confidence intervals (Fig. 1) supported this level of discrimination between sites and families. Receiver operating characteristic curves were between 0.95 and 1 (Table 1) which indicated the sensitivity of the QDFA was high.

**Table 1:** Classification success of the quadratic discriminant function analysis model comparing trace element:calcium ratios in adult *Perna canaliculus* from six families each grown at two sites in the Marlborough Sounds, New Zealand.

Actual site:family	Predicted site:family												Total n	% Correct site:family	% Correct site	% Correct family	ROC Curve
	1:A	1:B	1:C	1:D	1:E	1:F	2:A	2:B	2:C	2:D	2:E	2:F					
1:A	16	1	0	0	0	0	0	2	0	0	0	1	20	80	90	80	0.98
1:B	2	14	0	0	0	0	0	3	0	0	0	0	19	73.7	84.2	89.5	0.98
1:C	0	0	17	1	0	0	0	0	0	0	0	0	18	94.4	100	94.4	1
1:D	0	0	1	16	0	0	0	1	0	0	0	1	19	84.2	89.5	84.2	0.99
1:E	1	0	0	3	8	2	0	0	0	0	2	1	17	47.1	82.4	58.8	0.99
1:F	1	0	1	1	0	14	0	0	0	0	2	1	20	70	85	75	0.98
2:A	0	0	0	0	0	0	16	4	0	0	0	0	20	80	0	80	0.99
2:B	0	0	0	0	0	0	0	17	1	0	0	0	18	94.4	100	94.4	0.98
2:C	0	0	0	0	0	0	0	2	15	0	0	0	17	88.2	0	88.2	0.99
2:D	0	0	0	0	0	0	0	1	0	19	0	0	20	95	0	95	0.99
2:E	0	0	0	1	0	1	1	0	0	0	16	1	20	80	10	80	0.98
2:F	2	1	0	0	0	0	0	5	2	0	1	9	20	45	15	45	0.95
n Predicted	22	16	19	22	8	17	17	35	18	19	21	14	228	**77.6**	**91.7**	**80.3**	

Note the boxes indicate individuals grown at the same site.

**Table 2:** Cumulative classification success rates of the stepwise variable selection procedure on the QDFA comparing the trace element composition of adult *Perna canaliculus* from 6 families grown at two sites in the Marlborough Sounds, New Zealand.

*TE*:Ca Ratio	Cumulative % classified correctly		
	Site:Family	Site	Family
Co:Ca	24.1	54.3	29.3
Ti:Ca	42.5	75.8	46.9
Li:Ca	47.3	78.9	53
Sr:Ca	58.3	82.8	64
Mn:Ca	64	86.4	68.8
Ba:Ca	65.7	86.8	70.1
Mg:Ca	73.2	89.9	77.6
B:Ca	77.6	91.6	80.2
Pb:Ca	78.5	90.7	81.1
Cu:Ca	85	94.2	86.4
Ni:Ca	87.7	96	88.5

The Co:Ca ratio contributed to the greatest degree of classification with individuals correctly classified to their growth site with 54% accuracy, their family with 29% accuracy and their site:family combination with 24% accuracy using only this *TE*:Ca ratio (Table 2). The next most significant *TE*:Ca ratio was Ti:Ca which increased growth site classification to 73% (a 22% increase), family classification to 47% (a 17.6% increase) and site:family classification to 43% (an 18% increase) (Table 2).

#### Univariate statistics

**Figure 2:**
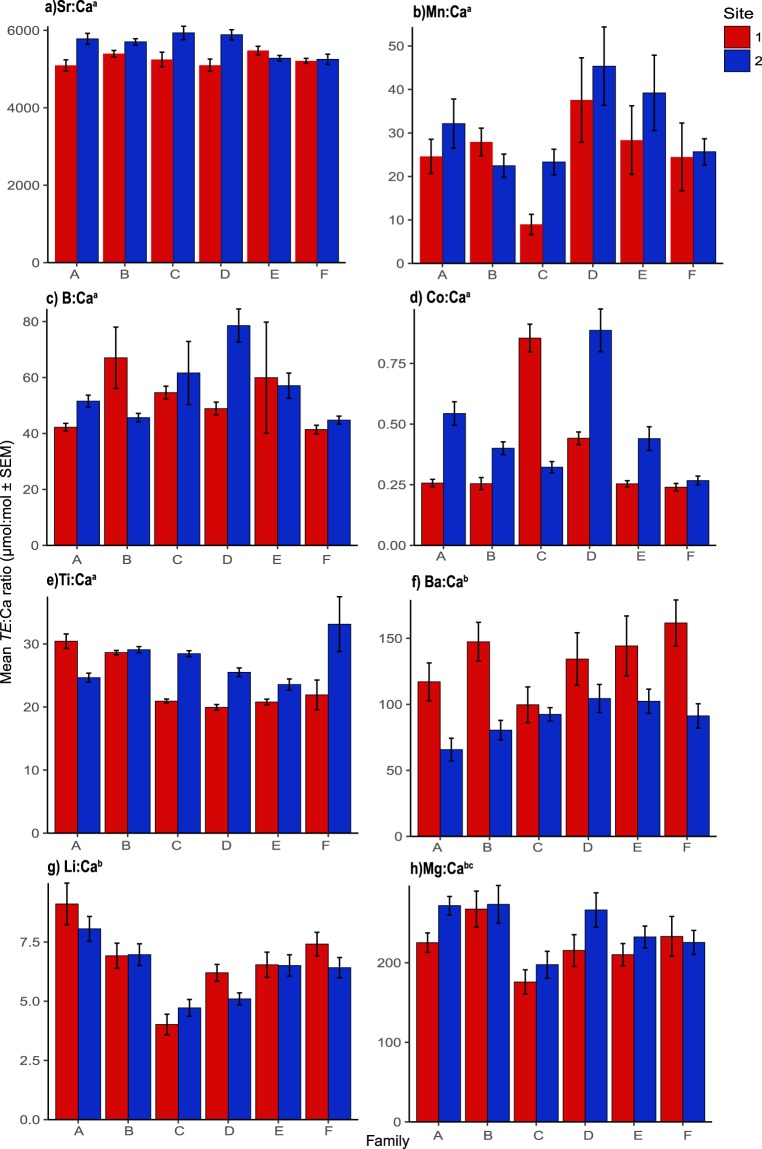
Mean trace element:calcium ratios (µmol:mol ± SEM) in the shell of six adult families of *P. canaliculus* cultured at the two different sites in the Marlborough Sounds, New Zealand. Note: Superscript (**a**) indicates GLM showed a significant family:site interaction effect, (**b**) indicates a significant main effect of site and (**c**) indicates a significant main effect of family.

The results of the GLMs investigating how *TE*:Ca ratios in the shell of *P. canaliculus* grown in the field indicated a significant interaction between site and family existed for Sr:Ca (F_5,226_ = 4.35, p < 0.01), Mn:Ca (F_5,226_ = 2.5, p = 0.03), B:Ca (F_5,226_ = 5.6, p < 0.01), Co:Ca (F_5,226_ = 22.32, p < 0.01), and Ti:Ca (F_5,226_ = 18.32, p < 0.01) ratios (Fig. 2a–e). Post-hoc pairwise t tests indicated that for all elements a number of within and between site significant differences were shown for these elements (Table S5). Significant main effects of site were found for Ba:Ca (F_1,227_ = 6.94, p = 0.01), Li:Ca (F_1,227_ = 17.1, p < 0.01) and Mg:Ca (F_1,227_ = 7.62, p = 0.01) (Fig. 2f–h). Pairwise comparisons of these elements showed that multiple families were responsible for these differences (Table S6). A significant main effect of family was found for Mg:Ca ratios (F_5,227_ = 6.94, p = 0.01) (Fig. 2h).

### Laboratory study

**Figure 3:**
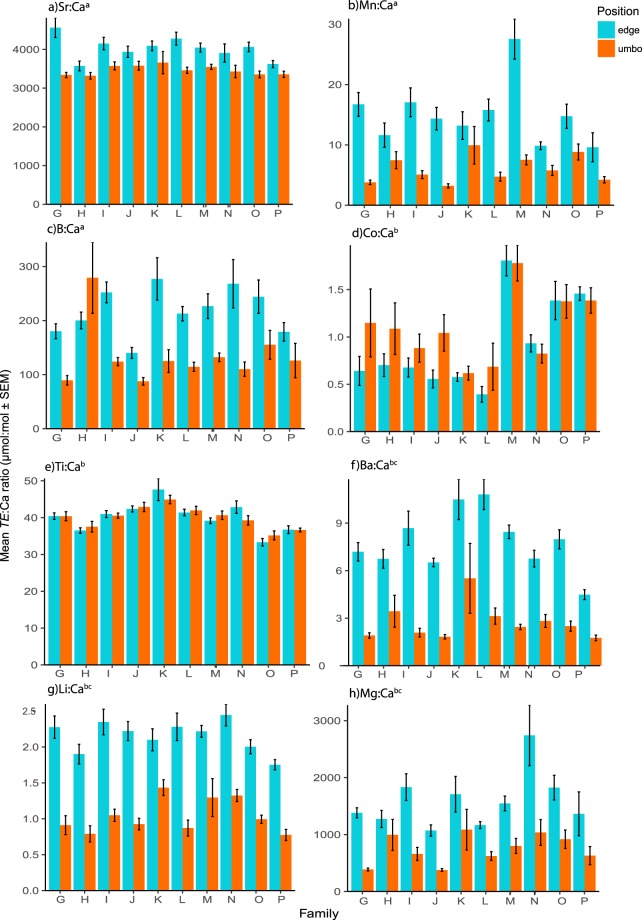
Mean trace element:calcium ratios (µmol:mol ± SEM) at each analysis position for each of the ten *P. canaliculus* families cultured under constant laboratory conditions. Note: Superscript (**a**) indicates GLM showed a significant family:position interaction effect, (**b**) indicates a significant main effect of family and (**c**) indicates a significant main effect of analysis position.

In laboratory cultured shells strontium was the most abundant element and cobalt was the least abundant (Table S7). Generalised linear models indicated that there were significant interaction effects between the position analysed within each shell and family for Sr:Ca (F_9,168_ = 2.1, *p* < 0.03), Mn:Ca (F_9,168_ = 4.45, *p* < 0.01), and B:Ca (F_9,168_ = 2.81, *p* < 0.01) ratios (Fig. 3a–c). Post-hoc comparisons showed that a number of family-location differences was driving this variation (Table S8) There was a significant main effect of family for Li:Ca (F_9,169_ = 2.9, *p* < 0.01), Mg:Ca (F_9,169_ = 5.16, *p* < 0.01), Ti:Ca (F_9,169_ = 16.09, *p* < 0.01), Co:Ca (F_9,169_ = 9.93, *p* < 0.01), Ni:Ca (F_9,169_ = 10.79, *p* < 0.01), Cu:Ca (F_9,169_ = 7.20, *p* < 0.01), Ba:Ca (F_9,169_ = 5.65, *p* < 0.01), and Mg:Ca (F_9,169_ = 7.9, *p* < 0.01) ratios. Multiple pairwise differences were shown to be responsible for these differences (Table S9). There was a significant main effect of the position analysed within shells for Li:Ca (F_1,168_ = 315.6, *p* < 0.01), Mg:Ca (F_1,168_ = 292.35, *p* < 0.01), and Ba:Ca (F_1,168_ = 925.44, *p* < 0.01) (Fig. 3). In all cases where significant differences were found to exist within shells the *TE*:Ca ratio at the edge was higher than that at the umbo.

## Discussion

This study demonstrates that trace elemental incorporation into calcified structures is a complex interplay between biological and environmental influences. We show that differences in the trace elemental composition of *P. canaliculus* shell can be caused by genetic variation between families, ontogeny, the environment, and interactions between these factors. Although previously demonstrated in fish otoliths^16–18^, this is the first time that the genetic history of individuals has been shown to affect bivalve shell chemistry. Despite differences in the trace elemental composition of shells between families, however, individuals were still able to be classified to the site at which they grew with a high degree of accuracy. In addition to the differences between families and the location at which individuals were grown, this study also demonstrated that the age of the animal at the time of shell formation may also affect the incorporation of trace elements into carbonate structures, supporting the findings of previous studies^12,14,19^.

### Are environmental differences between sites or genetic differences between families responsible for the trace elemental composition of *P. canaliculus* grown at two separate sites?

The composition of *P. canaliculus* shell differed both within and between sites in our *in situ* trial. This suggests both genetic and environmental differences factors are responsible for trace elemental fingerprints. Despite within site variation between families over 90% of individuals were correctly assigned to their growth location when analysed via QDFA. This suggests that although genetic variation between families will affect how trace elements are incorporated into shell, the environment still plays a dominant role. Such a finding is important as it shows that the use of elemental fingerprinting remains viable for connectivity studies even where gene flow is sufficient to maintain genetic homogeneity. This modulation of environmental effects by genetic factors may explain the difficulties in calibrating the trace elemental concentrations in shell material with environmental conditions^13–15^.

### Does the trace elemental composition of genetically distinct, *P. canaliculus* families vary when grown under identical laboratory conditions?

As shown in the laboratory study, in the absence of environmental differences the genetic history of an animal will influence the composition of bivalve shell. Complementary to the results of the *in situ* trial, these results are important as they indicate that even in the absence of environmental differences, genetically isolated stocks could be identified using elemental fingerprinting. This may allow this technique to be used in connectivity studies of species with very short pelagic larval durations that do not disperse over sufficiently large spatial scales that would incorporate environmental gradients. As these short distance dispersers are often overlooked this could provide insight into the role played by these organism in structuring ecosystems^21^. This result may also allow the use of trace elemental fingerprinting to delineate stocks over large areas where no environmental gradients exist.

### Potential drivers of variation in trace elemental fingerprints

Although the exact physiological mechanisms of biomineralisation are poorly understood, there are a number of possible explanations for the differences in trace elemental fingerprints between families and sites observed in this study. One possibility is the differences in biomineralisation processes are genetically driven and heritable. It has been shown with *Mytilus edulis* that over only a few generations shell structure may change in response to environmental variables indicating selection pressure can quickly modify biomineralisation processes^22^. The large variation in proteins responsible for biomineralisation within bivalves suggests that there may be a certain degree of plasticity in biomineralisation processes which allow adaptation to environmental changes^23,24^.

A second possibility is that physiological differences, which can be a product of both environmental and genetic variation^25^, may have contributed to observed differences in trace elemental fingerprints between families and sites. Growth rates have been demonstrated to affect the incorporation of elements into bivalve shell suggesting metabolic processes affect their incorporation into shell^13,14,26^. Elements which are transported to the site of biomineralisation through active intracellular Ca^2+^ pathways by Ca^2+^-ATPase will likely be more affected by variation in metabolic rates between families than those which are transported through passive pathways^27^. Although conflicting results exist, it has been suggested that the uptake of Mn, Zn, Cu and Sr are transported through active Ca^2+^-ATPase pathways^25,28^. This may explain the interactions observed between family and growth site for Sr and Mn due to the interaction between genetics and the environment moderating metabolism. Elements which are passively transported to the site of biomineralisation are more likely to reflect environmental variables in their shell chemistry. Barium in shell, for example, is thought to reflect barium levels in water and is transported through dietary pathways^6^ which may explain the differences observed in Ba:Ca ratios of shell grown at different sites and the lack of interaction effects found for this element.

These findings have reinforced the need to further understand biomineralisation processes. Understanding the pathways through which elements are transported to the site of biomineralisation will allow us to better delineate the role played by biology from that played by the environment in influencing trace elemental fingerprints in shells. As well as shedding light on biomineralisation processes, understanding the drivers of variation in trace elemental fingerprints will allow for the development of an optimum suite of elements to be used in situations where known environmental gradients exists. This will speed up analysis, reduce costs, and improve accuracy^29^. Additionally it may contribute to our ability to reconstruct the conditions at the time of shell formation^30^. The elements responsible for the classification of individuals to their growth site have all been previously used in trace elemental fingerprinting studies^29,31,32^. Cobalt, titanium, and lithium were responsible for the majority of this classification success. The factors driving the incorporation of cobalt and titanium into *P. canaliculus* shell are complex as shown by the interactions between different factors in both the laboratory and *in situ* study. However, there has been very little research into the factors affecting cobalt, lithium, and titanium relative to other elements. Further research into the factors affecting these elements in bivalve shell may allow the environmental and physiological controls on their incorporation into shell material to be better understood.

### As a mussel grows, what impact does this have on the trace elemental composition of its shell?

As shell material is generally accepted as being metabolically inert once deposited^19,33^ the two time points sampled in animals from the laboratory experiment present a record of conditions at the time of shell deposition. Therefore the differences observed within shells from the laboratory study where all environmental conditions were held constant demonstrates that ontogenetic changes affect how trace elements are incorporated. The fact that all elements investigated, with the exception of titanium were in some way affected by the age at which shell material was deposited indicates the important role played by ontogeny in shaping the trace elemental fingerprint of *P. canaliculus*. It has been suggested that changes in the trace elemental composition of CaCO_3_ structures as an individual ages can be due to cell aging resulting in a decreased ability to select Ca^2+^ ions; changes in metabolism as discussed earlier; reproductive status; or decreasing growth rates as individuals age resulting in shell material representing larger time scales being analysed^14,19^. Additionally, there is the possibility that ontogenetic changes in shell mineralogy and carbonate coordination will alter the ability of the CaCO_3_ matrix to accommodate certain trace elements^34,35^. Understanding how the elemental composition of bivalve shell is affected by age is essential for elemental fingerprinting studies. If the trace elemental composition of shell material varies at different ages even under identical conditions care needs to be taken when analysing shells by laser ablation to ensure that differences in the composition of shell material as an animal ages are not attributed to movement of an individual across an environmental gradient. It is also important to determine if spatial differences between sites are greater than within shell differences grown at these sites. If shell material is able to be assigned to its formation location regardless of age, the process of establishing a “reference atlas” of trace elemental fingerprints will be greatly simplified^32^ as shell material formed later in an individual’s life may be used as a proxy for that formed when an individual is older.

It is also possible that maternal condition and investment into the production of gametes beyond simple genetics may have affected the composition of shell material. Maternal investment can vary between individuals^36^ and it has been shown that larger larvae with more lipid reserves may be able to better maintain acid-base regulation^37^ and therefore control biomineralisation processes more tightly. This may explain some of the variation observed between families as well as between the two points analysed in shells. The effects of differential maternal investment would likely be clearer in shell material produced early in an individual’s life (at the umbo) particularly in the prodissoconch I shell which begins to form whilst *Perna canaliculus* larvae are still in a lecithotrophic stage^38^.

### Potential limitations

Despite the findings of this study, it is important to acknowledge possible limitations. Only two sites were investigated, which results in a much simpler QDFA model as significant variation in only one element between sites may be sufficient to correctly classify all individuals. Additionally, an *a priori* statistical technique which requires all potential source populations to be categorised was used. Although in this study all potential source populations were categorised, in population connectivity studies this is often impossible to achieve due to the multitude of possible sources. However, as this study shows that we may be able to estimate genetic variation from the trace elemental fingerprint of individuals, future studies should focus on the development of alternative statistical techniques, such as those proposed by White *et al*.^39^ or Neubauer *et al*.^40^.

Although we assume that differences in environmental parameters between sites are likely due to their geographic location, this study would have benefited from closer monitoring of these conditions. A number of studies however, have attempted to relate environmental conditions to the levels of trace elements in shells with varying levels of success^6,7,10–12^. Explicitly relating environmental differences to shell chemistry in this case would have been difficult due to complicated interactions between different environmental variables and trace elemental fingerprints. Finally, future studies investigating the effect of ontogeny in the shell of *Perna canaliculus* should examine multiple time points to determine if the differences observed in shell material deposited at different ages represents the beginning of a trend or if elemental incorporation stabilises as an individual ages.

## Conclusions

Overall, this research has provided additional insight into the factors which influence the incorporation of trace elements into bivalve shell. They provide more uses for fingerprinting over small scales. The findings that genetic variation and ontogeny influence the uptake of trace elements provides a possible explanation for the conflicting relationships between the environment and *TE*:Ca ratios which have been reported in the literature. These results have the potential to allow the further refinement of the technique of trace elemental fingerprinting to allow patterns of genetic connectivity to be unravelled through the analysis of bivalve shell. Finally, there is growing consensus that the integration of both genetic and trace elemental markers in carbonate structures is essential for accurate estimates of population connectivity to be obtained^41,42^. As these studies become more widespread there is an opportunity for further investigation into links between genetic differences between populations and their trace elemental fingerprint.

## Methods

### Animal Spawning and husbandry

#### *In situ* trial

Are environmental differences between sites or genetic differences between families responsible for the trace elemental composition of *P. canaliculus* grown at two separate sites?

Six F_2_ families from the Cawthron commercial *Perna canaliculus* selective breeding programme were investigated (Families A-F). Animals were spawned in 2014 and transported to two *in situ* sites where they were grown on long-line aquaculture facilities in the Marlborough Sounds, New Zealand. Each family was ongrown at two sites located approximately 10 km apart in Pelorus Sound. Site One was located in outer Pelorus Sound and therefore subject to more oceanic influences. Site Two was located in mid Pelorus sound and is therefore more affected by freshwater input and terrestrial influences. Twenty animals from each family at each site were harvested on the 8^th^ of April 2018, after a period of moderate rainfall^43^.The flesh was removed, shells were frozen at −20 °C and transported to the University of Auckland where they were stored at −20 °C until elemental analysis.

In preparation for elemental analysis each valve was scrubbed thoroughly with a household brush to remove any large adhering particles and biofouling organisms. A fragment of the growing shell edge measuring approximately 15 mm × 15 mm was broken off the left valve of each individual along the axis of maximum growth. This fragment was then rewashed in deionised water and mounted on a glass microscope slide using double sided adhesive tape. As the periostracum is not a calcified structure and is comprised primarily of organic matter it was removed through desiccation which caused it to peel off the shell.

#### Laboratory study

Does trace elemental composition of genetically distinct, *P. canaliculus* families vary when grown under identical laboratory conditions? Does ontogeny affect the trace elemental composition of shell material?

To examine differences in the trace elemental composition of shell in the absence of environmental differences and to determine the effect of ontogeny we performed a laboratory culturing experiment. Ten full-sibling families of *Perna canaliculus* were examined in this study (named hereafter Families G-P). Individual male and female mussels were randomly selected from unrelated F_2_ families raised on long-line aquaculture facilities in the Marlborough Sounds (South Island, New Zealand) as part of the Cawthron *Perna canaliculus* selective breeding programme. Thermal shock was used to induce spawning; eggs were diluted to 1000 mL^−1^ and fertilized with sperm at 200 egg^−1 ^^44^, and transferred to 160 L incubation tanks containing 5 µm-filtered seawater and 12 µM EDTA at 16 °C, with gentle aeration. After 48 h incubation the embryos had formed the prodissoconch I shell, entering the feeding veliger stage. Larvae were transferred to the continuous-flow culture system described by Ragg *et al*.^44^. Three 2.5 L tanks were used for each family. Density was approximately 200 larvae mL^−1^. They received filtered seawater enriched with dietary microalgae (*T-Isochrysis lutea* + *Chaetoceros calcitrans*, at a density of 40 × 10^6^ cells L^−1^). After 3 weeks, pediveligers were offered coir string as a settlement substrate and allowed to metamorphose and grow. Water properties including temperature, salinity, alkalinity and pH were monitored throughout the culturing phase (Table S1). Juveniles grew on coir string for a period of 90 days, after which they were harvested, rinsed in deionised water and snap frozen before being transferred to the University of Auckland where they were stored at −20 °C until analysis.

Prior to analysis shells were defrosted and rinsed thoroughly in deionised water. As they were cultured in a controlled system no biofouling organisms or adhering particles needed to be removed. One valve was selected at random from each individual and mounted whole on glass microscope slides using double sided adhesive tape. Due to the small size of the individuals it was not possible to remove the periostracum and a pre-ablation technique was used to ensure the periostracum was not included in reduced data which is described in the analytical methods section.

### Analytical methods

**Figure 4:**
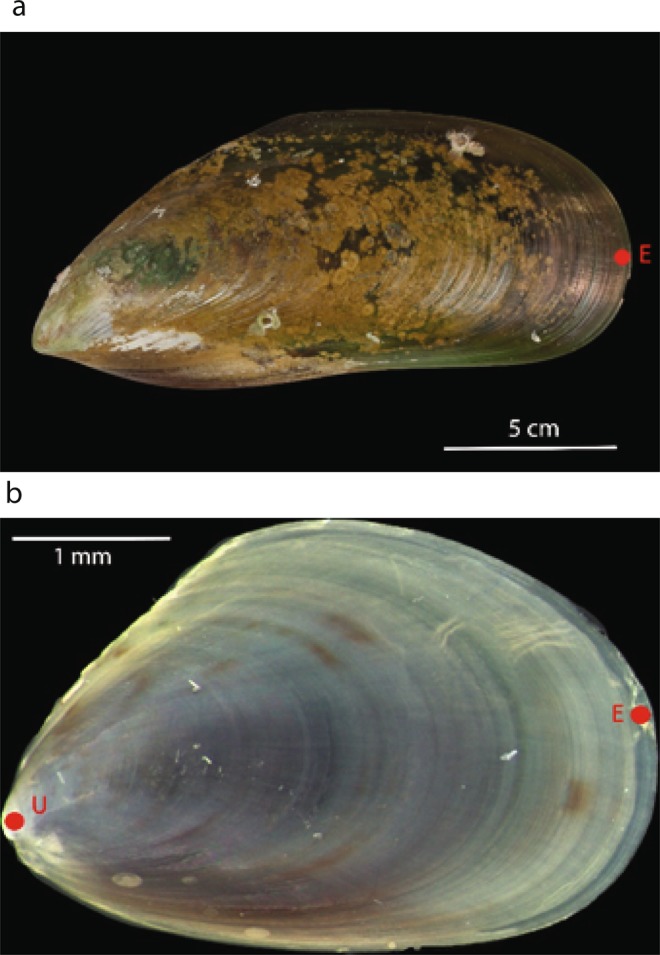
The locations at which LA-ICP-MS analyses were performed on example *P. canaliculus* shells shown with a red point (**a**) Location analysed on each of the adult shells (~4 years old) grown in the field. (**b**) Locations analysed on early juvenile shells (~90 days old) cultured in the laboratory. U represents the analysis at the umbo whilst E represents the analysis on the most recently formed shell edge.

To determine the trace elemental composition of shells we performed LA-ICP-MS analyses, using a New Wave deep ultra violet (193 nm) laser ablation system (Electro Scientific Industries) coupled to an Agilent 7700 ICP-MS (Agilent Technologies). One LA-ICP-MS spot analysis was performed on each of the shells grown *in situ* (*n* = 17–20 from each family:site combination). LA-ICP-MS analysis was performed 200 µm from the most recently formed edge (Fig. 4a). This region was selected in order to maximise the chances that shell material from all individuals formed within a relatively narrow time frame prior to harvest.

We analysed two positions within shells from the laboratory culturing experiments: one located approximately 200 µm from the most recently formed shell edge, and the other located on the shell umbo (Fig. 4b). These analysis positions were intended to represent shell formed within the first few weeks of an individual’s life and shell formed immediately prior to harvest. Where survival rates permitted, 10 shells were analysed from each tank (*n* = 30 per family); where less than 10 individuals were available as many individuals as possible from each family were analysed (n from each family is shown in Table S7).

Prior to each laser spot analysis, background element counts were monitored for 30 seconds. The laser operated with a spot size of 50 µm, a repetition rate of 5 Hz, and a dwell time of 40 seconds. Laser power was 45% and fluence was between 7 and 7.5 Jcm^−2^. National Institute of Standards and Technology (NIST) 610 and 612 glass standards were analysed every 20 spots for standardisation and calibration purposes. For full laser operating parameters see Tables S2 and S3. All analyses were performed at the University of Auckland Plasma Mass Spectrometry Centre.

To remove possible surface contamination we used a pre-ablation technique where the first five seconds of dwell time were not included in the data reduction process^12,45^. We used only the next 10 seconds of data to determine the elemental composition of the shell material in order to reduce the risk of laser burn through to lower layers within shell material^2^. We background-corrected data by subtracting background average counts from the ablation counts. Data was then standardised using the most recent published NIST610 values^46^ and NIST612 values were used to calculate internal precisions. All data was then standardised to a trace element:cacium ratio (*TE*:Ca) in µmol of trace element to mol of calcium.

### Statistical analyses

#### *In situ* trial

To determine how overall trace elemental fingerprints varied between sites and between families, as well as to determine if genetic variation prevented classification of individuals to their growth site, a quadratic discriminant function analysis (QDFA) was performed^20,41^. This was done stepwise by removing the least significant *TE*:Ca ratio whilst still maintaining site classification success rates of >90%. Receiver operating curves were then generated for each site:family combination to provide a measure of sensitivity of the DFA model. This QDFA was undertaken in JMP v13.0 (SAS institute, Carey, NC).

In order to determine how each of the elements responsible for classification varied, and to examine how they contributed to classification in the QDFA, univariate GLMs were performed in R v3.3.3 (R core team). Prior to analysis we inspected the data which did not meet the assumptions of normality, and we therefore log transformed the data which improved normality for all *TE*:Ca ratios investigated. The GLM examined the effect of growth site, family, and any interactions between these two terms. Post hoc t-tests were performed, with a false discovery rate (FDR) p-value adjustment applied due to multiple comparisons.

#### Laboratory study

To examine the effect of ontogeny and genetics on the incorporation of trace elements into *P. canaliculus* shell in the absence of environmental differences we performed univariate statistical analyses on the same *TE*:Ca ratios as above. Univariate analyses were performed in R v3.3. The data did not fit the assumptions of normality; thus, we performed a log transformation on all elemental ratios, which improved normality. We used additional GLMs to examine the effects of the burn position (shell edge vs umbo), family and any interaction effects. In the GLMs the three replicate tanks for each treatment were included as random factors. Post hoc t-tests were performed, with a false discovery rate (FDR) p-value adjustment applied due to multiple comparisons.

## Supplementary information

Supplementary material

## Data Availability

The datasets generated and during the current study are available in the figshare responsibility at https://auckland.figshare.com/articles/Trace_element_calcium_ratios_in_the_shell_of_Perna_canaliculus/7569008
